# Robotic-assisted surgery in the pediatric surgeons’ world: Current situation and future prospectives

**DOI:** 10.3389/fped.2023.1120831

**Published:** 2023-02-14

**Authors:** Hong Mei, Shaotao Tang

**Affiliations:** Department of Pediatric Surgery, Union Hospital, Tongji Medical College, Huazhong University of Science and Technology, Wuhan, Hubei, China

**Keywords:** robotic-assisted, robotic surgery, children, pediatric surgery, minimally invasive surgery

## Abstract

Robotic-assisted surgery has been fully embraced by surgeons for the adult population; however, its acceptance is too slow in the world of pediatric surgeons. It is largely due to the technical limitations and the inherent high cost associated with it. In the past two decades, indeed, there has been considerable advancement in pediatric robotic surgery. A large number of surgical procedures were performed on children with the assistance of robots, even with comparative success rates to standard laparoscopy. As a newly developing field, it still has many challenges and obstacles. This work is centered on the current status and progression of pediatric robotic surgery as well as the future perspectives in the field of pediatric surgery.

## Introduction

Laparoscopic procedures have been proven safe and valuable in adult and pediatric populations over the last few years. As previously shown, laparoscopy resulted in decreased hospital stays, smaller surgical scars, and expedited recovery (1). It even replaced open surgeries as the gold standard in some cases. However, its widespread use was impeded in more complex situations, particularly in intracorporeal anastomosis or extensive reconstruction. Laparoscopic procedures was difficult and largely related to the technological limitations with available laparoscopic instruments, 2D visualization, and a steep learning curve (2, 3). Additionally, infant and toddler patients pose a further challenge, for instance, narrow operative working space and quite delicate tissue mobilization in minimally invasive surgery (MIS) (4). With the introduction and application of robotic surgical platforms, a significant move has been made in the history of surgical evolution. Robots provide a magnified 3D view, more dexterity, high precision, and motion scaling, facilitating precise intracorporeal suturing and exposure (5). As a result of these advantages, this technology is adopted gradually for assisting pediatric surgeons in their surgical procedures. Its use has been applied widely, including pediatric urology, gastrointestinal surgery, and gynecology (6, 7). A growing number of reports have investigated its safety and efficiency in the pediatric population, compared with different approaches (open or laparoscopic surgeries). In this review, we are chiefly concerned with the available literature up to date, briefly surveyed the current status of robotic-assisted surgery in pediatrics, and critically analyzed its future development.

## Integration and history of robotics in pediatric surgery

da Vinci (Intuitive Surgical, Sunnyvale, CA, USA), the first robotic surgical system, was approved by FDA in 2000. It has been the only robotic surgery platform for a very long time and remains the predominant robotics system used worldwide. Despite preliminary studies showing that the conventional laparoscopic approach resulted in improved cosmesis and more efficiency compared with the open approach (8), the robot, instead, has revolutionized the concept of MIS. Since then, surgeons worldwide have embraced the rise of this new robotic surgery. The robotic platform provides many advancements, solving many tricky problems encountered during standard laparoscopy (9, 10). First, it has stable 3D visualization with 10 times magnification, and the camera is manipulated by the surgeon instead of the assistant. Second, it allows 7 degrees of freedom with uniquely designed endo-wrist instruments. The movements of robotic arms are not inverted, entirely different from traditional laparoscopy. Third, the ability to reduce tremors during movements and motion scaling is the best-known and more significant advantages. Finally, in stark contrast to laparoscopy, the robot offers surgeons better ergonomics to execute MIS procedures. Therefore, robotic surgery is currently a reality in surgical practice and has been widely accepted by the surgical field.

Many literature works reported that robotic surgery resulted in shorter hospital stays and equivalent measurable outcomes in the adult population, compared with conventional open or laparoscopic approaches (11, 12). This approach has become the gold standard in some selected procedures, such as prostatectomy. Just like the other new technology in surgery, pediatric robotic surgery is experiencing slow but steady development but still lagged far behind adult robotic surgery. This limitation is mainly because of the smaller working space of pediatric patients, and currently, no instrument with the appropriate size is available for them (13, 14). Therefore, at the initial attempts, practitioners cautiously selected teenagers to perform common robotic surgeries. In 2001, a case report describing a 10-year-old girl who underwent robotic Nissen fundoplication was the first report on this technology in pediatrics (15). Unlike extirpating in adult surgery, children usually require reconstructive procedures in most cases, which poses a further challenge, specifically in babies and infants. However, pediatric surgeons have always been adventurers and leaders in advancing surgical technology. When performing robotic surgeries, various tricks can be employed to overcome these limitations in pediatric patients (16, 17), such as placing ports to a more linear position with less triangulation, intussuscepting a 5-mm assistant trocar for suture introduction, performing aspiration and traction of the forth arms usually used in adults, and positioning pediatric patients with dedicated cushions and protecting with soft padding to prevent collision against the arms. Therefore, it appears that the robotic platform has enabled MIS in children to become mainstream in many complicated cases, which were previously difficult by laparoscopy, particularly in young children or infants (14). In recent years, pediatric robotic surgery has seen broader implementation and prevalence (see Table 1).

**Table 1:** Summary of the reported pediatric robotic surgical procedures in this review.

Urology surgery	General surgery	Cardiothoracic surgery	Other robotic surgeries
Pyeloplasty (19–23) Ureteral reimplantation (24–27) Partial nephrectomy (31) Nephroureterectomy (29, 30) Ureteroureterostomy (32) APV (33) Augmentation ileocystoplasty (34) Bladder neck reconstruction (35)	Nissen fundoplication (15, 36, 37) Cholecystectomy (38) Choledochocystectomy (40–46) Soave procedure for HD (47) Robotic surgery for ARM with a fistula (48) Repair of duodenal atresia (49) Duodenojejunostomy (50) Kasai operation for biliary atresia (51, 52) Hepatectomy (53, 54) Splenectomy (53) Robotic pancreatic procedures (55–59)	Diaphragmatic hernia (61) Lobectomy (62) Heller's myotomy (63) Esophageal atresia (64, 65)	Pediatric oncological surgery: Radical cystprostatectomy (66) Nephron-sparing surgery (67) Pheochromocytoma (68) Pediatric head and neck surgeries: Thyroid lobectomy (72) Neck surgery via a transaxillary or retroauricular approach (70, 71) Transoral robotic-assisted surgery (69)

## Distinctive robotic surgeries for pediatric patients

### Robotic urologic surgery in pediatric patients

The initial reported robotic urologic surgery in adults began with prostatectomy (18) and was subsequently followed by pediatric robot-assisted laparoscopic pyeloplasty (RALP), first accomplished in 2002 (19). Until 2015, about 40% of pyeloplasty surgeries on children were performed by robots in the United States (7). Several meta-analyses and systematic reviews of surgical outcomes after RALP have been reported. These data indicated that RALP achieved a shorter operative time (excluding docking time), decreased hospitalization, and a similar success rate to either open or laparoscopic procedures (20–23). Robotic ureteral reimplantation (RUR) is the second most common pediatric procedure with this technology, treated for vesicoureteral reflux (VUR), with a reported resolution rate of 77%–100% (24). Although it has been shown that there is a feasible selection for the laparoscopic technique through an extravesical or intravesical approach, RUR was largely performed by the extravesical (Lich-Gregoir) approach. There are some technical difficulties, such as trocar placement, pneumovesicum stability, and instrument navigation in small-capacity bladders, making intravesical RUR extremely challenging (25). Boysen and colleagues reviewed 260 patients from 9 centers who underwent extravesical RUR. The radiographic reflux resolution rate was 87.9%, with a total of 363 ureters, and the complication rate was low at 9.6%, in agreement with published data on open surgeries (26). Conversely, a 2016 study by Kurtz and colleagues compared 108 robotic vs. 1,494 open UR in 17 centers and demonstrated a higher complication rate in the robotic group (13.0% vs. 4.5%) (27). This difference might be related to case selection and the early learning curve of the operator (28). This approach is feasible for complete and partial nephrectomy or nephroureterectomy and has been reported by some study groups (29–31). However, it does not have any reconstructive phase, and we have a suspicion that the robot indeed offers a real advantage. Further multi-institutional and large-cohort studies are needed to assess which specific patients can benefit from robotic surgery.

Possibly because of the less steep learning curve of robotic surgery, it has subsequently been reported for more complicated reconstructive procedures in pediatric urology, including ureteroureterostomy, Mitrofanoff appendicovesicostomy (APV), bladder augmentation, bladder neck reconstruction, and augmentation ileocystoplasty (AI), among many others. Lee and colleagues retrospectively compared robotic to open ureteroureterostomy and concluded that there was a slightly shorter hospital stay in the robotic group, with no signification of operative times and complications (32). APV was traditionally carried out *via* open surgery, creating a continent tunnel in neurogenic bladder cases. Pedraza and colleagues described their successful experience of the first robotic APV in a 7-year-old boy with congenital posterior urethral valves (33). A small retrospective review, composing 17 robotic RAI and 13 open AI procedures, demonstrated that narcotic analgesia use, complication rate, and change in the bladder volume were similar between the two groups, while the median LOS was shorter in the RAI group (34). A more recent report by Adamic et al. included 24 patients who underwent RAI, of which 20 patients were successfully performed using da Vinci. A series of concomitant procedures were performed, including 16 APV, 8 antegrade continence enema (ACE), and 6 bladder neck reconstruction procedures (35). These results showed that the robot offers not only the intrinsic advantages of MIS but also comparable functional outcomes to open or laparoscopic procedures. It is reliable and effective for more complex procedures in children; however, we have to weigh the benefits of this technology against longer operation times and higher costs.

### Robotic general surgery in pediatric patients

Robotic general surgery in pediatric patients has been widely reported despite not reaching the magnitude of pediatric urology. Meininger and colleagues reported their robotic-assisted laparoscopic treatment of Nissen fundoplication in a girl, which was carried out in July 2000 and reported in April 2001; it was the first such case to be reported in a child (15). Since then, fundoplication has become one of the most widely performed robotic general surgeries in pediatrics (36). The published literature confirmed that it achieved similar results to conventional laparoscopic Nissen fundoplication in children (37). Although there are no obvious advantages compared with traditional laparoscopy, in this process, the operator can experience robotic surgical skills, and it is the best choice for pediatric surgeons who are performing robotic surgery for the first time. Cholecystectomies were carried out robotically in some institutes, but this robotic application has been questioned. Opponents believe that children cannot benefit more in this routine operation; on the contrary, it is associated with longer operating times and high costs (38). Single-site robotic cholecystectomy may have great appeal for the pediatric population and provide a potential cosmetic benefit through a novel robotic platform (39).

Excision of choledochal cysts in pediatric patients has been reported by several small cohorts undergoing robotic-assisted surgery (40–42) since first described in 2006 by Woo and colleagues (43). Because of its complexity during total cyst excision with Roux-en-Y reconstruction, open procedures are still relatively prevalent in many centers. A systematic review of robotic choledochocystectomy for children was performed by Wang and colleagues, including eight studies with an average age of 6.3 (0.3–15.9) years. Seventy-nine of 86 cases (91.9%) were successful, and seven patients (8.1%) experienced conversion. Ten patients (11.6%) had complications, including biliary leakage (8), wound disruption (1), and anastomotic stenosis (1, 44). They also investigated the proportion of methods used for intestinal anastomosis, with 54.6% of patients undergoing pull-through intestinal anastomosis and the remaining patients undergoing a complete robotic method. Both methods were feasible, mainly depending on the surgeon's preference. A recent study reported by Koga et al. revealed that total hepaticojejunostomy anastomotic time was significantly shorter and sutures were easier and more precise to handle with robotic equipment compared with conventional laparoscopic surgery (45). In addition, a better and magnified vision of the surgical field provides surgeons with clarity of the hepatic duct anatomy and far easier cyst excision and reconstruction of the biliary tree. Tang and his colleagues also retrospectively evaluated the outcomes in young infants (≤1 year) and control group (>1 years) and concluded that age did not hinder the successful implementation of this robotic surgery (46). These data indicate that robotic hepaticojejunostomy is practical and safe in children and can be considered an emerging approach.

There are relatively few reports on the application of robots in treating Hirschsprung's disease (HD) in children. The latest study was published in 2022, in which Quynh and colleagues analyzed 55 pediatric patients who underwent a robotic-assisted Soave procedure. They speculated that the shorter operative time might be related to experience and teamwork skills with procedural modifications (47). As a result of the advantages provided by robots, they might be helpful in a narrow space, especially in the small pelvic cavity of infants. Some case reports and series documented successful robotic surgery for anorectal malformations (ARMs) with a rectovesical or rectourethral fistula (48). A large cohort of 17 infants with ARMs attained favorable continence and defecation functions after robot-assisted anorectal pull-through (RAARP), which was suspected with minimal damage to perirectal nerves and external sphincters provided by the robotic system. There are also a few reports on robotic surgery for other neonatal diseases, such as the repair of duodenal atresia (49), duodenojejunostomy for SMA syndrome (50), and Kasai operation for biliary atresia. The first five robotic Kasai portoenterostomies were successfully completed (two by Dutta et al. (51) and three by Meehan et al. (52)) without perioperative complications, while the average time was quite longer than previous reports on open modality. The feasibility of robotic Kasai is still a subject of debate, and the long-term efficacy needs further verification (51, 52). Hepatectomy and splenectomy have been carried out robotically in children and have also been relatively prevalent in the literature (53). Rela and colleagues presented the first-ever report of robotic monosegment donor hepatectomy for liver transplantation in a 14-month-old girl with extrahepatic biliary atresia, highlighting the fact that it exhibited the maximum precision (54). Additionally, robotic surgery was increasingly being practiced in the treatment of pediatric pancreatic conditions, such as robotic spleen-preserving distal pancreatectomy (55) or robotic pancreatic enucleation (56) for the treatment of insulinoma, robotic lateral pancreaticojejunostomy for pancreatic duct stones (57), and robotic pancreatoduodenectomy (58) or partial pancreatectomy (59) for pancreatic tumors.

### Robotic cardiothoracic surgery in pediatric patients

Because of the smaller working space, pediatric cardiothoracic surgery is limited in the current literature. The most frequent robotic cardiothoracic procedures performed in pediatric patients are diaphragmatic hernia repair, lobectomy, bronchogenic cyst or mediastinal cyst excision, Heller's cardiomyotomy for achalasia, oesophagoplasty, and oesophageal atresia repair (60). Robotic repair of diaphragmatic hernia was successfully carried out in infants or even neonates using da Vinci (61). Durand et al. reported a series of lobectomies in children for treating severe bronchiectasis, comprising 7 robotic resections and 11 thoracoscopies (62). Altokhais et al. described robot-assisted Heller's myotomy in six patients for esophageal achalasia, ranging between 2 and 12 years, which might be a suitable alternative to MIS (63). Two cases of congenital esophageal atresia were successfully repaired using the da Vinci robotic system (64). A late study reported by Ferrero et al. revealed that esophageal robotic-assisted thoracoscopic surgery was performed for 18 patients, comprising 7 esophageal duplications, 4 esophageal atresias, 2 esophagoplasties, and 5 cases of Heller's myotomies. Two neonates (11%) needed a conversion due to exposure difficulties (65). It should be pointed out that the treatment of cardiothoracic diseases by the robot objectively exists the contradiction of large instruments and small thorax, the larger scar of skin puncture than that of a thoracoscope, and the significantly high cost (65). Robot cardiothoracic surgery in pediatric patients is still challenging; the indications should be strictly controlled, and only some selected patients are appropriate.

### Other robotic surgeries in pediatric patients

The application of robotics in pediatric oncological surgery was reported in some individual cases. Andenberg et al. reported the case of a 22-month baby with embryonal rhabdomyosarcoma of the bladder, who underwent the first robotic radical cystoprostatectomy (66). With excellent exposure for an oncological resection, Cost et al. presented a pediatric case of renal cell carcinoma, who received robotic nephron-sparing surgery combined with extensive lymphadenectomy (67). The robot could also permit concurrent partial resection of adrenal pheochromocytoma and total resection of extra-adrenal pheochromocytoma in a child with von Hippel–Lindau disease (VHL) (68). Indeed, as a minimally invasive approach, the robot in pediatric oncology surgeries has advantages, such as reduced narcotic analgesia usage, quick postoperative recovery, and low wound infection rate. The biology, treatment, and prognosis of tumors in children differ from those in adults. There is considerable disagreement over the suitability of the robotic approach extrapolated to the pediatric population from the adult literature data on robotic tumor surgery. Currently, robot usage for treating tumors in children is still low, but it is feasible and effective in carefully selected cases, and the application must comply with the oncological surgical principles.

More recently, robotic remote access has been adopted for head and neck surgeries *via* transaxillary, retroauricular, or transoral approaches, while it is slow in the pediatric population (69–72). In 2005, two teenage patients were first treated with a robotic transaxillary approach. One patient was scheduled for a right thyroid lobectomy, and the other was placed on a vagal nerve stimulator for treating intractable seizures (72). Wu and colleagues retrospectively reviewed pediatric patients (except one 20-year-old) who underwent robot-assisted neck surgery *via* a transaxillary or retroauricular approach and concluded that it was a feasible option for neck operations in the selected pediatric group in the hands of a vast majority of surgeons (70, 71). Erkul and colleagues reviewed the published literature on pediatric transoral robotic-assisted surgery and concluded that 90.2% of 41 patients could be completely treated robotically, and there was only 1 intraoperative complication (69). Robotic surgery is in the initial stage in the pediatric head and neck area, with limited experience. With increasing usage in head and neck surgery, the results will be encouraging in the future for pediatric robotic surgery.

## Limitations and future directions of robotic surgery

The main drawbacks remain the inherent high cost and technical limitations associated robotic surgery. The financial implications are associated with fixed costs (its relatively expensive prices and later maintenance cost) and variable costs of disposable usage. Compared to the large number of adult robotic operations, the number of pediatric operations is still very low, determining the high cost for the individual case. The only available da Vinci system has a stranglehold in the market with higher costs (73). Rowe and colleagues found that robotic surgery had an 11.9% reduction in direct expenses, mostly due to shorter hospitalization (74). They speculated that increased surgical volume and potential competitive market might ideally drive down indirect prices and even overall robotic surgery costs. In addition to the cost, there are other problems with applying da Vinci in the pediatric population, such as the size of robotic instruments (5 or 8 mm), technical limitations, learning curve, and inconvenience. In the past two decades, the robot has been continuously improved and upgraded several times (da Vinci Si, Xi, and SP). Several other distinctive robotic platforms are in various development stages, and even a few are already commercially available, such as Senhance Surgical Robotic System (75), Flex Robotic System (76), SurgiBot (77), and many new upcoming robots (73). The Senhance Surgical Robotic System provides 3-mm-sized instruments, which are particularly suitable for the younger children and neonates (75). However, so far, these new technical refinements have not been extended to pediatric cases. Examination of emerging robotic platforms should focus on the possibility of the future of pediatric robotic surgery specifically.

## Conclusion

Although this once seemed far away, pediatric robotic-assisted surgery has become part of the reality of surgeons. As the adoption becomes more widespread, its safety and effectiveness in children have been demonstrated, and the indication is growing steadily across other pediatric surgical subspecialties. However, the benefits of this technique over traditional laparoscopic or open approaches need to be truly assessed through further robust prospective investigations. The costs and technical constraints with the available robotic platform and instruments are the greatest hurdle, deterring its use in the pediatric population specifically. The innovation of robot technology will never stop. Robotic surgery in children will undoubtedly increase with the development of new platforms, miniaturization of instruments, and reduction of inherent costs.
